# Electrolyte disturbances after allogeneic hematopoietic cell transplantation in patients receiving calcineurin inhibitors: incidence and severity

**DOI:** 10.3389/fphar.2026.1934354

**Published:** 2026-09-14

**Authors:** Maria Clara Ferreira Camargo, Luiz Carlos da Costa-Junior, Marina Izu, Rita de Cássia Barbosa da Silva Tavares, Simone Cunha Maradei Pereira, Elias Hallack Atta, Maria Claudia Rodrigues Moreira, Marta Colares Nogueira, Décio Lerner, Teresa de Souza Fernandez, Paulo Caleb Júnior Lima Santos

**Affiliations:** 1 Department of Pharmacology, Escola Paulista de Medicina, Universidade Federal de São Paulo (EPM-Unifesp), São Paulo, Brazil; 2 Bone Marrow Transplant Center, Instituto Nacional de Câncer (INCA), Rio de Janeiro, Brazil; 3 Postgraduate Program in Medical Sciences, Faculdade de Medicina da Universidade de São Paulo (FMUSP), São Paulo, Brazil; 4 Bone Marrow Transplant Program, Hospital Universitário Clementino Fraga Filho (HUCFF), Universidade Federal do Rio de Janeiro (UFRJ), Rio de Janeiro, Brazil; 5 Cytogenetic Laboratory, Cell and Gene Therapy Program, Instituto Nacional de Câncer (INCA), Rio de Janeiro, Brazil; 6 Faculty of Pharmaceutical Sciences, Federal University of Alfenas, Unifal-MG, Minas Gerais, Brazil

**Keywords:** allogeneic HCT, arrhythmias, calcineurin inhibitors, electrolyte disturbances, GVHD

## Abstract

**Background:**

Electrolyte disorders are highly prevalent in critically ill patients and have been associated with severe clinical manifestations, including cardiac arrhythmia and increased mortality. In the context of allogeneic hematopoietic cell transplantation (HCT), graft-versus-host disease (GVHD), acute kidney injury (AKI), and the use of emetogenic conditioning regimens, calcineurin inhibitors, amphotericin B, foscarnet, diuretics, among other drugs, may contribute to the development of complex and interrelated electrolyte disturbances. However, studies addressing the incidence, severity, and clinical relevance of these electrolyte imbalances in allogeneic HCT recipients remain scarce. In addition, interindividual variability in calcineurin inhibitor exposure, partially influenced by pharmacogenetic factors such as *CYP3A5* polymorphisms, may contribute to differences in susceptibility to these disturbances.

**Objectives:**

To determine the frequency and severity of electrolyte disturbances in allogeneic HCT recipients and to evaluate potential pharmacogenetic-related factors associated with these alterations.

**Methods:**

This retrospective, single-center study included 374 patients who underwent allogeneic HCT and received tacrolimus or cyclosporine for graft-versus-host disease prophylaxis. Electrolyte disturbances involving potassium, sodium, magnesium, and calcium were defined according to laboratory reference values and graded using the Common Terminology Criteria for Adverse Events (CTCAE) version 5.0. Clinical and transplant characteristics were obtained from electronic medical records.

**Results:**

Electrolyte disturbances were highly prevalent, particularly those characterized by low serum concentrations, including hypocalcemia (86.6%), hypomagnesemia (85.6%), hyponatremia (75.7%), and hypokalemia (62.6%), whereas hypercalcemia was rare. Most disturbances were mild to moderate, although hypocalcemia, hyperkalemia, and hypokalemia accounted for a substantial proportion of severe cases. Electrolyte abnormalities frequently occurred concomitantly, demonstrating strong interdependence, especially among potassium, sodium and calcium. Acute kidney injury, total body irradiation, age, and concomitant electrolyte disturbances were identified as significant factors associated with these outcomes. No significant differences in the incidence or severity of disturbances were observed between tacrolimus and cyclosporine.

**Conclusion:**

Electrolyte disturbances after allogeneic HCT are common, often multifactorial, interdependent, and clinically relevant. These findings highlight the need for integrated monitoring and early management of electrolyte abnormalities in the post-transplant period.

## 1 Introduction

Hematopoietic Cell Transplantation (HCT) is a well-established cellular immunotherapy employed in the treatment of various malignant hematological diseases and certain benign conditions affecting the population. Between 2012 and 2024 in Brazil, 14,331 bone marrow transplants were reported across 45 Brazilian centers (Simione et al., 2025).

Electrolyte disorders are alterations in electrolyte levels found in bodily fluids that appear more frequently in children, due to physiological immaturity, and in the elderly, due to greater susceptibility to pathologies (Alexim, 2024). Several factors can lead to electrolyte imbalance, such as the use of certain medications, infections, neoplasms, as well as endocrine, genetic, autoimmune, gastrointestinal, renal, cardiac and pulmonary diseases (Alexim, 2024). Electrolyte disorders are highly prevalent in critically-ill patients; a recent study evaluating a cohort of patients admitted to an intensive-critical unit demonstrated that 31.3% presented at least one disorder upon admission, while 38.2% presented multiple disorders. Furthermore, 77.4% of patients developed at least one new disorder during hospitalization. There is also evidence to suggest that certain electrolyte disorders are frequently associated with alterations in other electrolyte levels, indicating a concomitant occurrence of disturbances (Bachmann et al., 2025). Outside the hospital setting, considering the general population, significant incidence rates of electrolyte disorders have also been recorded. In a study conducted with elderly individuals in the community, 15% presented at least one disorder, which was associated with diabetes mellitus, diuretic use, and an increased risk of mortality (Liamis et al., 2013).

Graft-versus-host disease (GVHD) is an immune-mediated complication of allogeneic transplantation that frequently affects the gastrointestinal tract, causing inflammation, mucosal injury, and diarrhea. These manifestations result in significant fluid and electrolyte losses, in addition to malabsorption, which contributes to the development of electrolyte disorders in post-transplant patients (Pereira et al., 2020).

Calcineurin inhibitors (tacrolimus and cyclosporine) are key components of prophylactic regimens indicated for GVHD (Funke et al., 2023). In the context of kidney transplantation, the use of these inhibitors to prevent organ rejection has been associated with the occurrence of various electrolyte and metabolic disorders (Lee and Kim, 2007; Tainio, 2018; Higgins et al., 2004). However, few studies have explored the significance of these electrolyte alterations in the HCT setting.

Calcineurin inhibitors have already been strongly associated with hypertension, dyslipidemia, and the development of post-transplant diabetes, factors that significantly contribute to a higher cardiovascular risk in transplant patients (Farouk and Rein, 2020). Electrolyte disturbances such as hypercalciuria and hypomagnesemia are known adverse effects resulting from tacrolimus action, and a significant decrease in renal mRNA expression of TRPV5, calbindin-D (28K), and TRPM6 (proteins involved in the active reabsorption of calcium and magnesium) is one of its main molecular mechanisms responsible for these effects (Nijenhuis et al., 2004).

Tacrolimus and cyclosporine, besides being associated with clinical outcomes such as GVHD and AKI, are also frequently associated with renal tubular dysfunction and electrolyte disturbances, including abnormalities in serum magnesium, potassium, and sodium levels (Naesens et al., 2009).

Electrolyte abnormalities have been shown to contribute to cardiac arrest, in addition to hindering resuscitation efforts (American Heart Association, 2000). Among electrolytes, potassium imbalances are the ones that can generate the most immediate and potentially fatal consequences. Physical symptoms of hyperkalemia include ECG changes which, if not properly treated, cause progressive cardiac dysfunction, resulting in weakness, ascending paralysis, and respiratory difficulty. Conversely, hypokalemia manifests itself through symptoms such as weakness, fatigue, paralysis, respiratory difficulty, rhabdomyolysis, constipation, paralytic ileus, and leg cramps. Furthermore, hypokalemia aggravates the toxic effects of digitalis and must be rapidly treated in patients using these derivatives (American Heart Association, 2000).

In a study developed by Lee et al., severe metabolic abnormalities and electrolyte disorders in allogeneic HCT were significantly related to inferior clinical outcomes. This study demonstrated that patients with at least one metabolic abnormality presented lower 5-year overall survival and significantly higher 100-day non-relapse mortality when compared to patients without severe abnormalities (Lee et al., 2005).

The impacts that electrolyte disorders can generate on the clinical status of transplant patients are extensive and may require immediate interventions. Although the incidence and severity of these alterations have already been described in patients undergoing autologous HCT (Philibert et al., 2008), studies addressing this topic in allogeneic transplants are still scarce. Thus, we conducted a retrospective, single-center investigation aimed at determining the frequency and severity of electrolyte disorders in allogeneic HCT recipients, as well as evaluating clinical and transplant-related factors potentially associated with these alterations.

## 2 Methods and patients

This was a retrospective, single-center study conducted at the Bone Marrow Transplantation Center of the *Instituto Nacional de Câncer* (INCA), Rio de Janeiro, Brazil. A total of 374 patients who underwent allogeneic hematopoietic cell transplantation (HCT) between 2010 and 2023 and were treated with calcineurin inhibitors (tacrolimus or cyclosporine) for GVHD prophylaxis were included.

Eligibility criteria included the following: the patient underwent allogeneic transplantation during the study period, used tacrolimus or cyclosporine as a calcineurin inhibitor, and had laboratory and clinical data available in electronic medical records. Patients were excluded from the study if they matched any of the following criteria: they switched calcineurin inhibitors, i.e., from cyclosporine to tacrolimus or tacrolimus to cyclosporine within the first 21 days, underwent syngeneic (twin) transplantation, lacked data availability for variable collection or only had data in physical records, their source of hematopoietic stem cells was umbilical cord blood.

The study was approved by the Research Ethics Committee of the *Instituto Nacional de Câncer* (INCA), Brazil (approval number 6.596.351), in accordance with national regulations for research involving human subjects.

### 2.1 Definition of electrolyte disturbances

Electrolyte disturbances were diagnosed as follows: hypokalemia (serum potassium <3.5 mmol/L), hyperkalemia (serum potassium >5.5 mmol/L), hypomagnesemia (serum magnesium <1.6 mg/dL), hypermagnesemia (serum magnesium >2.6 mg/dL), hypocalcemia (serum calcium <8.3 mg/dL), hypercalcemia (serum calcium >10.6 mg/dL), hyponatremia (serum sodium <136 mmol/L), and hypernatremia (serum sodium >145 mmol/L). The severity of each clinical condition was classified according to the Common Terminology Criteria for Adverse Events (CTCAE) version 5.0 (2017) (National Cancer Institute, 2017), with grades 1, 2, 3 and 4 corresponding to mild, moderate, severe and very severe events, respectively. To facilitate the presentation of results, grades were subsequently grouped into grades 1–2 (mild and moderate disturbances) and 3–4 (severe disturbances).

Electrolyte disturbances were evaluated during the first 21 days following initiation of calcineurin inhibitor therapy. For patients receiving conventional GVHD prophylaxis, this corresponded to days +1 to +21 after transplantation. For haploidentical transplant recipients, in whom calcineurin inhibitor therapy was initiated on day +5, the observation period corresponded to days +5 to +25. All available electrolyte measurements obtained during the respective observation period were reviewed, and patients were classified as having an electrolyte disturbance if at least one measurement met the CTCAE v5.0 criteria for the corresponding abnormality.

### 2.2 Clinical characteristics and outcome definition

Clinical and transplant characteristics were collected from medical and laboratory records, from which demographic data, underlying diagnosis of transplant recipients, conditioning regimen intensity, hematopoietic stem cell source, and GVHD prophylaxis regimen used were obtained. Data such as Cytomegalovirus (CMV) diagnosis in recipients and the need for ATG (Anti-thymocyte Globulin) therapy, Total Body Irradiation (TBI), and corticosteroid therapy (>2 mg/kg) were also previously collected.

The incidence of Acute Kidney Injury (AKI), including its moderate and severe stages, was determined according to the criteria established by Kidney Disease: Improving Global Outcomes (KDIGO) 2012 (Kidney Disease: Improving Global Outcomes KDIGO Acute Kidney Injury Work Group, 2012). For diagnosis, a relative increase in serum creatinine ≥1.5 times the previously recorded baseline value was adopted as a reference.

### 2.3 Statistical analysis

Clinical and transplant variables were presented as absolute and relative frequencies for categorical variables and as median ± SD for continuous data. To evaluate the association between clinical variables and electrolyte disorders, Chi-square tests were employed, with results presented in the form of tables and heatmaps. These analyses served as a screening tool to select variables to be applied in other statistical test models (univariate and multivariate logistic regression).

Association analyses between electrolyte disorders were performed using R software (version 4.4.2) with *broom*, *dplyr*, *tidyr* and *ggplot2* packages. For each pair of disorders, the OR and 95% confidence interval were estimated by binomial logistic regression. Multiple Correspondence Analysis (MCA) between electrolyte disorders (K, Na, Mg, Ca) was performed using *FactoMineR*, *factoextra* and *ggplot2* packages (R software) and presented as a plot.

In the univariate and multivariate logistic regression analyses, the occurrence of each electrolyte disorder was analyzed as a dichotomous variable (present/absent). To investigate potential associated factors, univariate logistic regression was performed for each outcome, estimating odds ratios (OR), 95% confidence intervals (95% CI), and p-values. Variables with p ≤ 0.20 in the univariate analysis were considered eligible for the multivariate model. Furthermore, variables clinically recognized as potential confounders were forced into the adjusted models (age, sex, transplant type, conditioning regimen, and calcineurin inhibitor use).

Independent multivariate models were adjusted for each outcome. Variables included in each model were selected according to significance in the univariate analysis and clinical relevance. For hyperkalemia - hypocalcemia, hypomagnesemia, hyponatremia and race/skin color were included; for hypokalemia - hypernatremia, AKI, hypermagnesemia, hypocalcemia and TBI; for hypermagnesemia - AKI and hyperkalemia; for hypomagnesemia - pediatric age and hypocalcemia; for hypernatremia - hypokalemia, AKI and hypermagnesemia; and for hyponatremia - age, TBI, hyperkalemia, corticosteroid use, hypocalcemia and race/skin color. In the hypocalcemia model - age, hyperkalemia, hypokalemia, hyponatremia, HCT source, hyper/hypomagnesemia, TBI and calcineurin inhibitor were included. For hypercalcemia - age, sex, HCT type and conditioning type were included. These latter variables were added to all models as they are potential confounding factors. For each adjusted model, OR, 95% CI, and p-value were reported.

Goodness-of-fit was evaluated by the Hosmer-Lemeshow test, and discriminative ability by the area under the ROC curve (AUC). The events-per-variable (EPV) ratio was inspected, with particular attention to values < 10.

P-values were two-tailed, with a significance level of 5% (p < 0.05) adopted for the interpretation of results. All analyses were performed using R software (version 4.x).

## 3 Results

### 3.1 Cohort characteristics: clinical and HCT

Of the 374 allogeneic HCT recipients, the majority were adults (67.1%) and male (58.8%). Regarding the underlying diagnosis, the majority presented malignancies, notably acute lymphoblastic leukemia (32.1%) and acute myeloid leukemia (25.1%). Acute Kidney Injury (AKI), an outcome assessed within the first 3 weeks after calcineurin inhibitor initiation, was diagnosed in 171 (45.7%) patients, with 28.9% of these already presenting moderate/severe grades (KDIGO 2–3) of this clinical condition.

Of the total patients, 55.6% were conditioned with a myeloablative regimen and 44.4% with a reduced-intensity regimen. On day 0, 70.1% of the patients received bone marrow grafts, while 29.9% received peripheral blood hematopoietic cells. Regarding HLA compatibility, nearly half of the transplants (44.9%) were from related donors, with 33.7% being from unrelated donors. However, a significant portion of patients (21.4%) received hematopoietic stem cells from haploidentical donors. Further details on demographic and clinical transplant characteristics are described in Table 1.

**TABLE 1:** Demographic and clinical characteristics of transplantation and incidence of acute kidney injury (AKI) at 21 days (N = 374).

Characteristics	Total (374)	
	N	%
Age range		
Adult	251	67.1
Pediatric	123	32.9
Receiver age (median, SD)	28.2 ± 18.4	
Sex receptor		
Feminine	154	41.2
Masculine	220	58.8
HCT type		
Related	168	44.9
Haplo	80	21.4
Unrelated	126	33.7
Receiver weight (median, SD)	62 ± 23.2	
Color or race		
White	178	47.6
Brown	150	40.1
Black	46	12.3
HCT source		
Bone marrow	262	70.1
Peripheral blood	112	29.9
Diagnosis		
AML	94	25.1
CML	40	10.7
ALL	120	32.7
MDS-MPN	40	10.7
BMF	51	13.6
Lymphomas-MM	29	7.8
CMV receptor		
Positive	349	93.3
Negative	25	6.7
ATG		
Yes	158	42.2
No	216	57.8
Conditioning regimen		
Myeloablative	208	55.6
Reduced intensity	166	44.4
TBI		
Yes	186	49.7
No	188	50.3
GVHD prophylaxis		
CSA/MTX	239	63.9
TAC/MMF/PTCy	37	9.9
TAC/MTX	33	8.8
CSA/MMF/PTCy	65	17.4
Corticoid (>2 mg/kg)		
Yes	174	46.5
No	200	53.5
CNI		
CSA	304	81.3
TAC	70	18.7
Cyclophosphamide		
Yes	251	67.1
No	123	32.9
Amphotericin B		
Yes	47	12.6
No	327	87.4
AKI (21 days)		
Yes	171	45.7
No	203	54.3
AKI - KDIGO 2–3 (21 days)		
No	266	71.1
Yes	108	28.9

Abbreviations: HCT, hematopoietic cell transplantation; CSA, ciclosporine; TAC, tacrolimus; GVHD, graft versus host disease; PTCy, post transplant cyclophosphamide; MMF, micophenolate mofetil; MTX, methotrexate; BMF, bone marrow failure; AML, acute myeloid leukemia; ALL, acute lymphoblastic leucemia; CML, cronic myeloid leukemia; MDS/MPN, myelodysplastic syndromes and myeloproliferative neoplasms; MM, multiple myelome; CMV, cytomegalovirus; AKI, acute kidney injury; KDIGO, Kidney Disease: Improving Global Outcomes; ATG, Anti-thymocyte Globulin; TBI, total body irradiation.

### 3.2 Incidence, severity and clinical factors associated with electrolyte disturbances

Analysis of Figure 1 demonstrates that electrolyte disturbances were highly prevalent, with a higher incidence of disorders characterized by low serum electrolyte concentrations, namely, hypocalcemia (86.6%), hyponatremia (75.7%), hypomagnesemia (85.6%) and hypokalemia (62.6%), while episodes of hypercalcemia were rare. The analysis comparing the incidence and severity of disturbances between the two calcineurin inhibitors used showed no statistically significant differences between them. This result can be observed in Figure 2.

**FIGURE 1:**
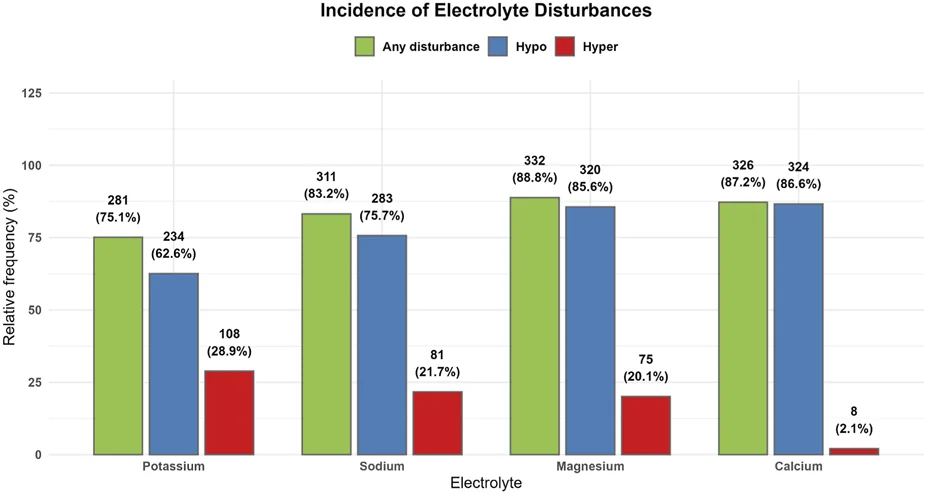
Incidence of electrolyte disturbances by electrolyte. Bar chart presenting the absolute and relative frequencies of electrolyte disturbances observed in the first 21 days after the start of immunosuppressants (tacrolimus and cyclosporine). Each group of bars represents a specific electrolyte (potassium, sodium, magnesium, calcium) and includes three categories: “Any disturbance” (light green), “Hypo” (blue) and “Hyper” (red). The “Any disturbance” category corresponds to patients who presented at least one episode of electrolyte imbalance (hypo or hyper) during the observation period. It is important to highlight that the same patient may have presented both hypo and hyper for the same electrolyte within the 21-day interval, which means that the frequency of “Any disturbance” may be higher than the sum of the isolated frequencies of hypo and hyper.

**FIGURE 2:**
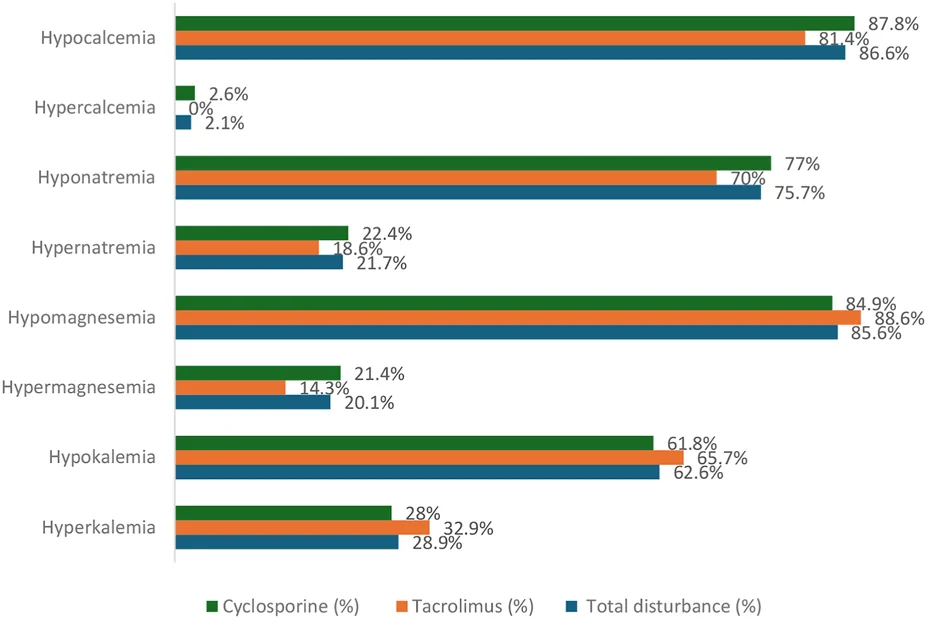
Frequency of electrolyte disturbances within 21 days of starting immunosuppressants, according to calcineurin inhibitors (N = 374).

Among the disturbances, grades 1 and 2 were more frequently observed (Figure 3), with only hypocalcemia, hyperkalemia and hypokalemia accounting for a more significant percentage of grade 3 and 4 severities (20.6%, 12% and 24.1%, respectively).

**FIGURE 3:**
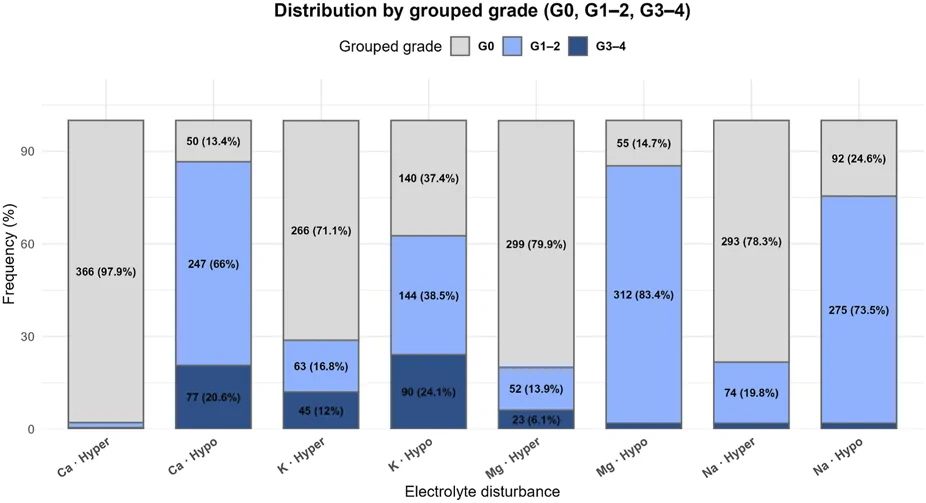
Distribution of the degrees of electrolyte disturbances (G0, G1–2, G3–4). Bar chart presenting the percentage distribution of severity grades of electrolyte disturbances observed among patients during the first 21 days after the initiation of immunosuppressants (tacrolimus and cyclosporine). Each bar represents an electrolyte disturbance (K, Na, Mg, Ca), subdivided by alteration types, hypo and hyper, indicating, respectively, hypo- and hyperelectrolytemia. The grades were grouped into three main categories: G0 (absence of disturbance), G1–2 (mild to moderate disturbances), and G3–4 (severe disturbances). Only values with a proportion ≥4% were labeled with the absolute count and the corresponding percentage. Unclassified cases were represented in light gray.

Figure 4 demonstrates that electrolyte disturbances tend to occur concomitantly, suggesting a positive correlation between them at varying magnitudes. It is worth noting that calcium disorders were predictors for the development of all other disturbances, whereas magnesium disorders were predictors only for the occurrence of calcium disorders.

**FIGURE 4:**
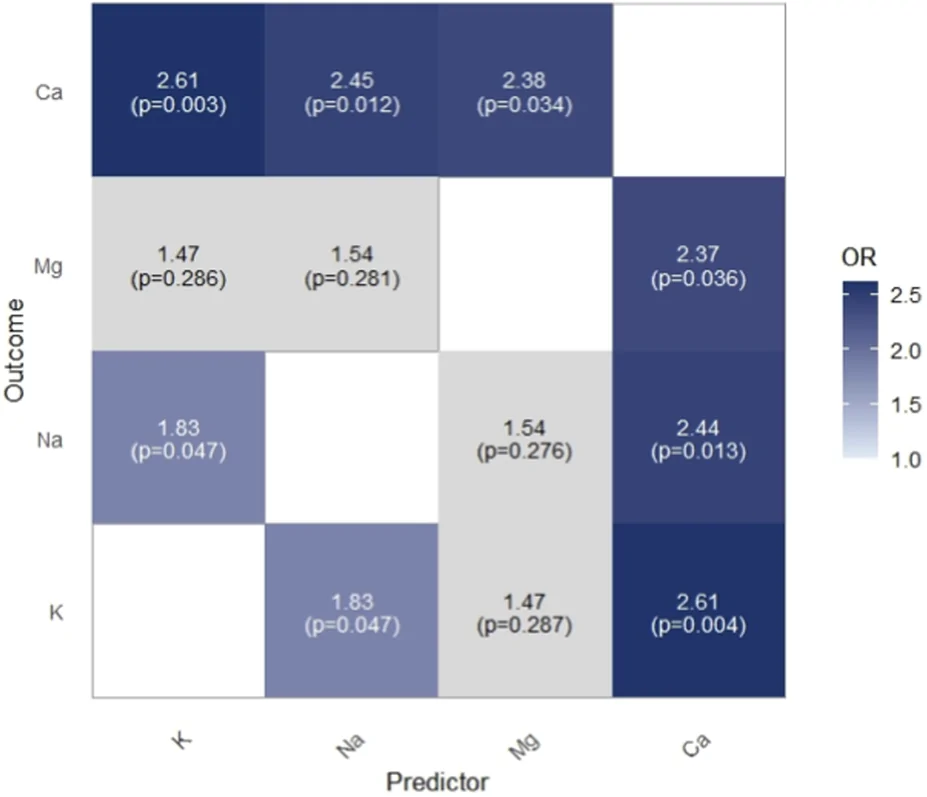
Association between electrolyte disturbances. Cross-associations between potassium (K), sodium (Na), magnesium (Mg), and calcium (Ca) disturbances in hematopoietic stem cell transplant recipients within the first 21 days after the start of immunosuppressant treatment. Each cell of the matrix represents an unadjusted binary logistic regression, where the disturbance on the vertical axis was considered the outcome and that on the horizontal axis the predictor. The values inside the cells indicate the odds ratio (OR) and the respective p-value. Cells in shades of blue indicate statistically significant associations (p < 0.05), while those in gray correspond to non-significant associations (p ≥ 0.05).

Figure 5 presents the Multiple Correspondence Analysis (MCA) of the binary variables representing the presence (=1) or absence (=0) of potassium, sodium, magnesium, and calcium disturbances. The first two dimensions explained 58.4% of the total variability (Dim1 = 35.3% and Dim2 = 23.1%). Categories corresponding to the presence of electrolyte disturbances (K = 1, Na = 1, Ca = 1, and Mg = 1) clustered closely on the left side of the plot, whereas categories representing their absence (K = 0, Na = 0, Ca = 0, and Mg = 0) were more dispersed on the right side. This spatial distribution indicates a tendency for electrolyte disturbances to occur simultaneously rather than independently. The strongest pattern of co-occurrence was observed among potassium, sodium, and calcium disturbances, while magnesium disturbance also clustered with these variables, reinforcing the overall interdependence among electrolyte abnormalities.

**FIGURE 5:**
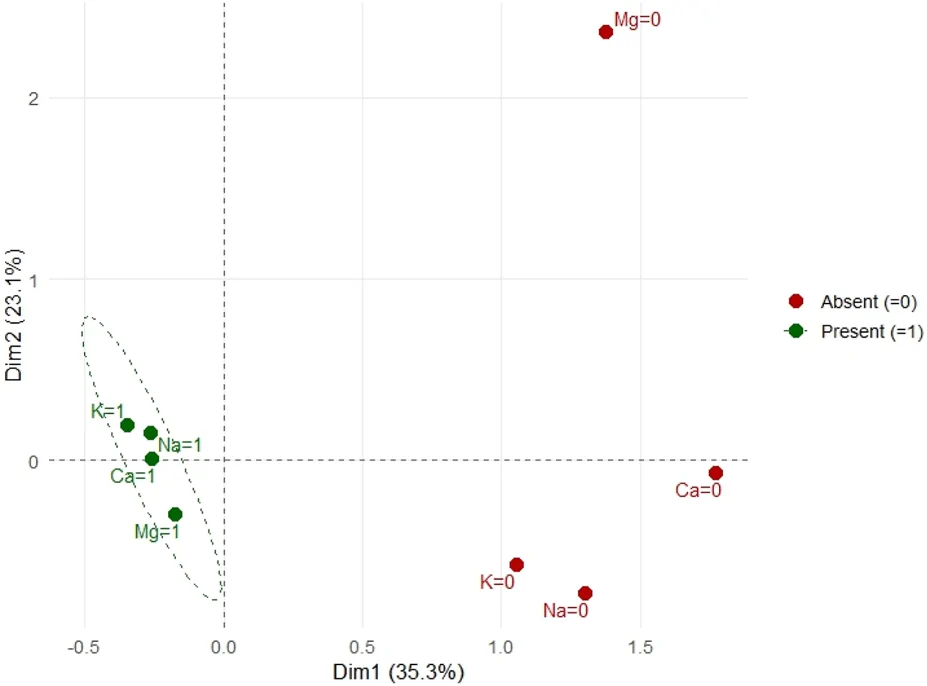
Multiple Correspondence Analysis (MCA) between electrolyte disturbances (K, Na, Mg, Ca). Multiple Correspondence Analysis (MCA) of electrolyte disturbances. Distribution of the presence (=1, green) and absence (=0, red) of potassium (K^+^), sodium (Na^+^), magnesium (Mg^2+^), and calcium (Ca^2+^) disturbances. The first two dimensions explained 58.4% of the total variability (Dim1 = 35.3%; Dim2 = 23.1%). Categories representing the presence of electrolyte disturbances clustered together, indicating a tendency toward co-occurrence, whereas categories representing their absence were more dispersed.

Figure 6 provides an integrated overview of the statistically significant associations identified by the chi-square analysis between clinical variables and electrolyte disturbances. The principal findings highlighted in the heatmap were: (a) an association between hyperkalemia and hypocalcemia; (b) grade 2–3 acute kidney injury (AKI) and hypernatremia associated with hypokalemia; (c) age, age group (adult vs. pediatric), and total body irradiation (TBI) associated with hyponatremia; (d) grade 2–3 AKI associated with hypermagnesemia; and (e) age, age group, and hyperkalemia associated with hypocalcemia. Darker color intensities represent lower p-values and therefore stronger statistical evidence of association.

**FIGURE 6:**
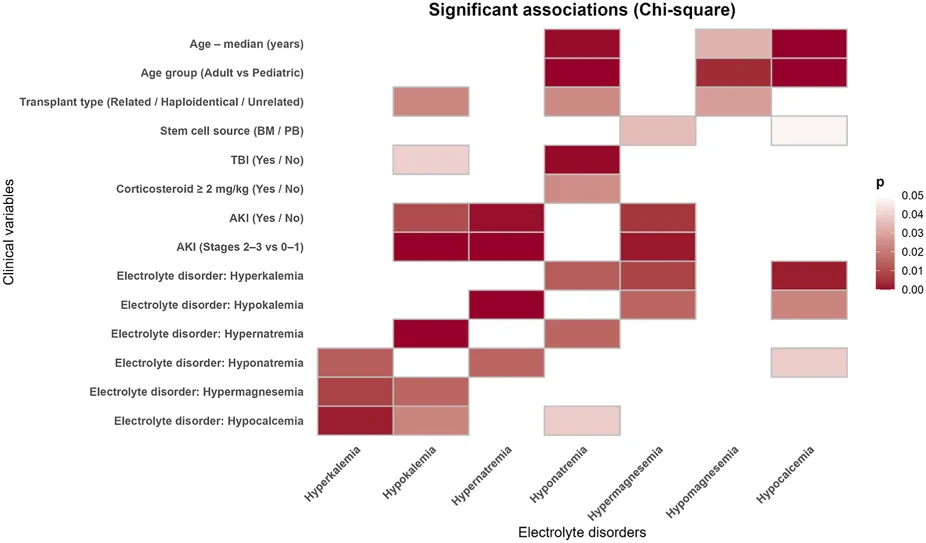
Significant associations between clinical variables and electrolyte disturbances after hematopoietic stem cell transplantation. The heatmap represents the statistically significant associations identified by the Chi-square test between selected clinical variables (vertical axis) and electrolyte disturbances (horizontal axis). Each colored cell indicates a comparison where there was a significant difference in the distribution of categories. Color intensity is proportional to the p-value, ranging from dark red (lower p-values, stronger associations) to white (p-values close to 0.05). Only comparisons with a calculated p < 0.05 were included. The variables were organized to maintain the correspondence between “hyper” and “hypo” pairs of the same electrolyte (potassium, sodium, magnesium and calcium), allowing for direct visualization of the opposite directions of electrolyte alteration.

### 3.3 Potassium disturbances

Hypokalemia was observed in 62.6% of patients (Figure 1), with 38.5% diagnosed with grades 1–2 of the disorder and 24.1% with grades 3–4 (Figure 3). This complication showed a significant association in the Chi-square test with: HCT type variables (related, haploidentical, unrelated); TBI (yes vs. no); occurrence of hypermagnesemia, hypocalcemia and hypernatremia; and AKI (stages 2–3 vs. 0–1). The association with the latter two was strongly positive, with p-values <0.01 (Figure 6). However, after multivariable analysis (Table 2), only AKI (stages 2–3) and hypernatremia remained significantly associated with the outcome, indicating that the occurrence of these factors increased the likelihood of a patient belonging to the hypokalemia group. Although total body irradiation (TBI) was identified as a risk factor for hypokalemia in the univariate analysis, this association lost statistical significance in the adjusted model, suggesting that its effect does not remain independently associated with hypokalemia after controlling for other covariates.

**TABLE 2:** Univariate and multivariate logistic regression.

Uni and multi logistic regression						
Outcome	Variable	OR (uni) (IC 95%)	P (uni)	OR (adj) [IC 95%]	P (adj)	HL
Hyperkalemia	Hypocalcemia	4.19 (1.77–12.38)	0.003	3.57 (1.45–10.80)	0.011	0.849
	Hypermagnesemia	2.03 (1.19–3.44)	0.008	1.89 (1.08–3.28)	0.024	
	Hyponatremia	2.06 (1.17–3.80)	0.015	1.94 (1.06–3.69)	0.036	
	Color/race (black vs. white)	2.08 (1.06–4.07)	0.032	2.36 (1.16–4.81)	0.017	
Hypokalemia	Hypocalcemia	2.00 (1.09–3.65)	0.024	1.76 (0.90–3.50)	0.101	0.159
	AKI (stages 2–3 vs. 0–1)	2.90 (1.75–4.97)	<0.001	2.95 (1.43–6.19)	0.004	
	Hypermagnesemia	2.00 (1.15–3.62)	0.017	1.69 (0.91–3.20)	0.101	
	Hypernatremia	5.01 (2.64–10.35)	<0.001	4.26 (2.15–9.14)	<0.001	
	TBI (yes vs. no)	1.56 (1.02–2.38)	0.040	1.74 (0.97–3.17)	0.065	
	HCT type (haplo vs. related)	2.21 (1.25–4.03)	0.008	2.02 (0.97–4.29)	0.064	
Hypernatremia	AKI (stages 2–3 vs. 0–1)	3.23 (1.93–5.41)	<0.001	2.24 (1.03–5.18)	0.049	0.908
	Hypokalemia	5.01 (2.64–10.35)	<0.001	3.90 (2.00–8.23)	<0.001	
	Color/race (black vs. white)	1.87 (0.86–3.90)	0.102	2.33 (1.00–5.28)	0.045	
Hyponatremia	Hypocalcemia	1.93 (1.01–3.61)	0.041	1.10 (0.52–2.26)	0.804	0.085
	Age (adult vs. pediatric <18 anos)	0.34 (0.21–0.56)	<0.001	0.33 (0.13–0.76)	0.011	
	Hyperkalemia	2.06 (1.17–3.80)	0.015	2.14 (1.16–4.14)	0.018	
	Color/race (brown vs. white)	0.59 (0.35–0.98)	0.042	0.65 (0.37–1.13)	0.125	
	Color/race (brown/black vs. white)	1.75 (1.08–2.86)	0.025	1.61 (0.95–2.77)	0.078	
	TBI (yes vs. no)	0.36 (0.22–0.60)	<0.001	0.46 (0.24–0.87)	0.018	
Hypermagnesemia	Graft source (PB vs. BM)	0.52 (0.27–0.94)	0.038	0.53 (0.26–1.01)	0.061	0.852
	AKI (stages 2–3 vs. 0–1)	2.35 (1.38–3.97)	0.001	1.89 (0.90–4.20)	0.104	
	Huperkalemia	2.03 (1.19–3.44)	0.008	1.96 (1.14–3.38)	0.015	
Hypomagnesemia	Hypokalemia	2.00 (1.15–3.62)	0.017	1.93 (1.03–3.74)	0.045	0.235
	Age (adult vs. pediatric <18 anos)	0.43 (0.24–0.77)	0.004	0.28 (0.09–0.76)	0.016	
	HCT type (haplo vs. related)	0.43 (0.22–0.87)	0.019	0.49 (0.24–1.03)	0.059	
Hypercalcemia	HCT type (unrelated vs. related)	4.15 (0.94–28.66)	0.085	6.07 (1.28–44.08)	0.036	0.736
Hypocalcemia	Age (adult vs. pediatric <18 anos)	0.30 (0.16–0.54)	<0.001	0.97 (0.31–2.94)	0.958	0.054
	Hyperkalemia	4.19 (1.77–12.38)	0.003	3.98 (1.57–12.27)	0.007	
	Hypokalemia	2.00 (1.09–3.65)	0.024	2.06 (1.05–4.09)	0.037	
	Hyponatremia	1.93 (1.01–3.61)	0.041	0.99 (0.47–2.04)	0.988	

Abbreviations: HCT, hematopoietic cell transplantation; BM, bone marrow; PB, peripheral blood; AKI, acute kidney injury; TBI, total body irradiation; OD, odds ratio.

Hyperkalemia developed in 28.9% of patients (Figure 1), with 16.8% diagnosed with milder and moderate forms of the disorder (Grades 1–2) and 12% with more severe forms (Grades 3–4) (Figure 3). In the univariate analysis, hypocalcemia, hypermagnesemia, hyponatremia, and Black race/skin color were significantly associated with an increased likelihood of belonging to the hyperkalemia group. After multivariable adjustment, all of these variables remained independently associated with the outcome, indicating that the occurrence of previous electrolyte balance disturbances involving calcium, magnesium, and sodium, as well as being of Black race/skin color, consistently contribute to an increased chance of the patient belonging to the group of individuals who develop hyperkalemia (Table 2).

Throughout the study, 67.1% of the patients received cyclophosphamide, either as part of the conditioning regimen (pre-transplant) or as prophylaxis for GVHD (post-transplant). In addition, 12.6% of the patients received amphotericin B for the prevention of fungal infections. Although several reports in the literature describe associations between cyclophosphamide use and the incidence of hyponatremia, as well as between amphotericin B use and the incidence of hypokalemia, these associations were not statistically significant in the present study.

### 3.4 Sodium disturbances

Hyponatremia was a disorder observed in 75.7% of patients (Figure 1), with the majority (73.5%) diagnosed with grades 1–2 (Figure 3). In the Chi-square analysis (Figure 6), low sodium concentrations were shown to be significantly associated with variables such as age, HCT type, TBI treatment, corticosteroid treatment (>2 mg/kg), and the occurrence of hyperkalemia, hypernatremia and hypocalcemia. After adjusted logistic regression analysis, adult age group, hyperkalemia, and treatment with total body irradiation (TBI) were identified as factors associated with an increased likelihood of belonging to the hyponatremia group (Table 2).

Hypernatremia was observed in 21.7% of patients (Figure 1), with 19.8% diagnosed with grades 1–2 of the disorder (Figure 3). The occurrence of hypernatremia was shown to be associated with the clinical variables AKI (stages 2–3 vs. 0–1), hypocalcemia, and hyponatremia (Figure 6). However, after univariate logistic regression analysis (Table 2), only the presence of AKI (stages 2–3) and hypokalemia were shown to be significantly associated with the outcome. In other words, the development of AKI (stages 2–3) and hypokalemia independently increases the patient’s chance of belonging to the group of individuals who develop hypernatremia. Nevertheless, it is important to highlight that, after adjustment of the analysis, the magnitude of the associations was reduced, suggesting that part of the effect was explained by confounding variables.

### 3.5 Magnesium disturbances

Hypomagnesemia was observed in 85.6% of patients (Figure 1), with 83.4% diagnosed with grades 1–2 of the disorder (Figure 3). Low serum magnesium concentrations showed a significant association with patient age and the HCT type (related, haploidentical, unrelated) that they underwent (Figure 6). In the univariate analysis, the presence of hypokalemia, adult age group, and haploidentical transplantation were shown to be factors that increase the patient’s chance of belonging to the group of individuals who develop hypomagnesemia. However, after the adjusted analysis, the latter variable lost statistical significance and, therefore, was not considered an independent effect (Table 2).

Hypermagnesemia occurred in 20.1% of patients (Figure 1), with 13.9% diagnosed with grades 1–2 of the disorder and 6.1% with higher severity grades (Grades 3–4) (Figure 3). In the univariate analysis, peripheral blood graft source, the presence of acute kidney injury (AKI) stages 2–3, and hyperkalemia were shown to be factors that increase the patient’s chance of belonging to the group of individuals who develop hypermagnesemia. However, after controlling covariates (adjusted analysis), only the association with hyperkalemia remained, indicating that alterations in this electrolyte independently contribute to an increased chance of belonging to this group (Table 2).

### 3.6 Calcium disturbances

Hypocalcemia was present in 86.6% of patients (Figure 1), with 66% diagnosed with grades 1–2 of the disorder and 20.6% with higher severity grades (Grades 3–4) (Figure 3). In the univariate analysis, adult age group and the presence of hyperkalemia, hypokalemia, and hyponatremia were shown to be factors that increase the patient’s chance of belonging to the group of individuals who develop hypocalcemia. However, after controlling covariates (adjusted analysis), only potassium imbalances (hyperkalemia and hypokalemia) were shown to be independent factors for this increased chance (Table 2).

Finally, evaluating disorders characterized by high serum calcium concentrations, which were present in only 2.1% of patients (Figure 1), it was observed in the univariate analysis that unrelated HCT type did not behave as a factor that significantly increases the patient’s chance of belonging to the group of individuals who develop hypercalcemia. However, after multivariable adjustment (Table 2), the magnitude of the association of this variable (OR) increased and became significant, indicating that confounding variables were attenuating this effect in the crude analysis.

## 4 Discussion

The findings of this study show the complex pathophysiological interaction among multiple electrolyte disturbances observed in patients undergoing HCT, highlighting consistent associations between alterations in sodium, potassium, magnesium and calcium. Furthermore, the clinical consequences of the observed dysfunctions were explored, such as the role of hyperkalemia and hypokalemia in the genesis of severe arrhythmias, and the relevance of hypomagnesemia as a central element in both the maintenance of hypokalemia and the development of hypocalcemia. Thus, this body of evidence supports the idea that such disorders occur in an interrelated manner and share common physiological mechanisms, underscoring the importance of an integrated approach in the assessment and management of these patients.

Significant associations were found in the study between potassium and dysnatremias (hyperkalemia vs. hyponatremia, and hypokalemia vs. hypernatremia). Adequate distal delivery of water and sodium is one of the essential factors for the control of renal potassium secretion. This occurs because the greater the distal sodium delivery, the greater the stimulus for its absorption, which renders the lumen potential more negative and, consequently, favors potassium secretion (Palmer, 2015). Calcineurin inhibitors, by blocking this phosphatase, allow the phosphorylation of kinases such as SPAK and WNKs, which are responsible for activating NCC transporters (sodium-chloride cotransporter). When activated, these transporters increase sodium chloride reabsorption in the distal convoluted tubule, which results in decreased sodium delivery to more distal segments. Thus, there is a decrease in the electrical gradient favoring potassium secretion, hindering this process (increased serum potassium–hyperkalemia) (Pochineni and Rondon-Berrios, 2018). Cyclosporine has also been shown, in *in vitro* studies, to reduce Na+, K+-ATPase activity in cortical collecting tubules, which impairs potassium secretion and sodium reabsorption (Tumlin and Sands, 1993; Deppe et al., 1997).

Contrary to the findings of the present study, where there were no significant differences in the incidence and severity of disturbances between tacrolimus and cyclosporine, a study developed by Higgins et al. found that hyponatremia and hyperkalemia were more frequent in renal transplant recipients treated with tacrolimus than cyclosporine, indicating differences in nephrotoxicity induced by the two drugs. However, this study also highlighted associations between the occurrence of sodium and potassium disorders, as patients with hyponatremia presented a higher probability of developing hyperkalemia, which aligns with our findings (Higgins et al., 2004).

Another factor caused by calcineurin inhibitors that could explain the concomitant incidence of hyperkalemia and hyponatremia lies in their potential to affect the renin-angiotensin-aldosterone system. These drugs can inhibit the transcription of mineralocorticoid receptors, causing their downregulation, which culminates in mineralocorticoid dysfunction and aldosterone resistance that may be accompanied by clinical pictures of hypoaldosteronism, even with normal plasma aldosterone levels (Pochineni and Rondon-Berrios, 2018; Heering et al., 2004; Deppe et al., 2002). A case series study, which highlighted a pediatric patient who underwent hematopoietic stem cell transplantation, and two others who underwent renal transplantation, demonstrated that these patients, who were under treatment with calcineurin inhibitors, presented decreased potassium excretion and increased sodium excretion (hyponatremia and hyperkalemia). The possible cause of the disturbances was attributed to hyporeninemic hypoaldosteronism, and treatment with fludrocortisone proved effective (Unsal et al., 2024).

The observed association between hypomagnesemia and hypokalemia can be explained by well-established physiological mechanisms. Intracellular magnesium plays a fundamental regulatory role in renal potassium secretion via the ROMK channel, located in the apical membrane of distal nephron cells. Under normal conditions, intracellular Mg^2+^ binds to the interior of the channel, promoting its inward rectification and limiting K^+^ efflux into the tubular lumen. When magnesium depletion occurs, this inhibition is lost, resulting in increased tubular potassium secretion. Notably, this mechanism explains the refractoriness of serum potassium correction when magnesium is not restored, something that is frequently described in patients with hypomagnesemia secondary to calcineurin inhibitor use, diuretics, or tubular dysfunction in transplant recipients. It is important to emphasize that hypomagnesemia alone is not sufficient to cause hypokalemia (Aboghanem and Prasad, 2024; Huang and Kuo, 2007).

Although it was not a significant association in this study, it is important to highlight that hypocalcemia is also a common manifestation of hypomagnesemia, which could explain, for instance, that the coexistence of hypomagnesemia and hypocalcemia with potassium disorders, within the context cited above, is physiologically plausible and clinically expected in this patient group. Magnesium deficiency impairs the release of PTH (parathyroid hormone), which is essential for regulating blood calcium levels (increases calcium reabsorption in the kidneys, promotes calcium absorption in the intestine, and stimulates calcium release from bones into the blood). Furthermore, it has been described that hypomagnesemia helps promote increased PTH metabolism and target-organ resistance to its action (Swaminathan, 2003).

A factor widely described in the literature that may be associated with the high incidence of hyponatremia among patients in the present study is the use of cyclophosphamide, a drug extensively employed in conditioning regimens preceding transplantation and as prophylactic therapy for GVHD (Bruining et al., 2011). Cyclophosphamide-induced hyponatremia has been mainly observed following high-dose administration (Bonella and Warley, 2017); however, this electrolyte disturbance has also been reported after the use of lower doses and even after a single oral dose of cyclophosphamide (Lee et al., 2010; Gilbar et al., 2012). The primary mechanism described as the cause of cyclophosphamide-induced hyponatremia is the drug’s ability to induce a syndrome characterized by excessive release of antidiuretic hormone (ADH), known as the syndrome of inappropriate antidiuretic hormone secretion (SIADH) (Krishnamurthy et al., 2020).

Hyperkalemia is the electrolyte disturbance that is most associated with the development of potentially fatal arrhythmias and cardiorespiratory arrest. These cases can be observed through ECG abnormalities, which vary as a function of both the absolute level of serum potassium and the rate of its increase. When hyperkalemia is untreated, the QRS complex observed on the ECG fuses with the T wave to form a sine wave pattern with imminent cardiac arrest. As serum potassium rises, more than one ECG abnormality may be present, such as a sine wave pattern, QRS widening, ventricular tachycardia, and bradycardia, factors that further increase the risk of cardiac arrest (Alfonzo et al., 2006).

Considered the most common disturbance in hospitalized patients, hypokalemia can also predispose patients to arrhythmias and cardiac arrest. Potassium and/or magnesium depletion is strongly associated with the malfunction of ion channels responsible for ventricular repolarization. Thus, it can cause arrhythmias such as *torsade de pointes*, which may manifest as syncope or cardiac arrest. Furthermore, hypokalemia can potentiate the toxicity of antiarrhythmics and interfere with the effects of these medications, potentially leading to underlying arrhythmias as well (Alfonzo et al., 2006). The use of amphotericin B as antifungal prophylaxis is a highly prevalent therapy in clinical practice in HCT settings, a factor that may be associated with the high incidence of hypokalemia observed among patients in the present study (Liberatore et al., 2021). A study published in 2020 involving hemato-oncological patients who were intermittently treated with high doses of liposomal amphotericin B demonstrated that hypokalemia was the most common grade 3–4 adverse event observed across prophylaxis cycles (Youngs et al., 2020).

As mentioned previously, hypomagnesemia is also associated with the development of significant cardiovascular manifestations. Clinical trials have demonstrated a reduction in mortality and fewer arrhythmias in patients with acute myocardial infarction who received magnesium (Ahmed and Mohammed, 2019). Moreover, magnesium depletion has been shown to be associated with atherosclerotic disease, as low levels of this electrolyte cause endothelial dysfunction, a hypercoagulable state, and increased lipid deposition, in addition to potentially causing coronary artery hyperreactivity to vasoconstrictor stimuli (Ahmed and Mohammed, 2019). Studies evaluating the relationship between magnesium and cardiovascular risk in patients with chronic kidney disease (CKD) have demonstrated that peritoneal dialysis patients who developed arterial calcifications presented significantly lower serum magnesium levels (Meema et al., 1987).

Most disturbances in patients in the present study were those characterized by low electrolyte concentrations. A possible explanation for these events would be the occurrence of a “genesis syndrome” (rapid uptake), characterized by a massive and rapid transfer of electrolytes into proliferating cells (Anandan et al., 2020). A study evaluating the dynamics of electrolytes such as phosphorus and potassium in multiple myeloma patients undergoing autologous hematopoietic stem cell transplantation demonstrated that engraftment occurred at a median of 10 days, and the median time for these electrolytes to reach their nadirs was 10 and 4.28 days, respectively, indicating a significant association between the magnitude of the electrolyte drop and the marked increase in cell count during the engraftment period (Anandan et al., 2020). Another study developed by Faghihi et al. also demonstrated a decreasing pattern in electrolyte concentration, with nadir values preceding the recovery of leukocyte and platelet counts (Faghihi et al., 2009).

According to Hierlmeier et al., there are early complications that are common after hematopoietic stem cell transplantation (HSCT) in pediatric patients. It is known that one of the most severe complications associated with allogeneic HSCT is GVHD. According to Hierlmeier, 59.5% of their pediatric study patients developed acute GVHD, while 22.2% presented chronic GVHD (Hierlmeier et al., 2018). Also in our results, we observed that 46.5% of patients were treated with corticosteroids (>2 mg/kg) to control established GVHD.

Kidney damage may also be present soon after HSCT, mainly in response to medications such as immunosuppressants, anti-infectives and chemotherapeutic agents (Kersti et al., 2007; Liu et al., 2007). In our study, 45.7% of patients developed Acute Kidney Injury in the first 21 days of immunosuppression, and of these, 28.9% presented moderate/severe grades of this clinical condition.

Although pharmacogenetic variables were not evaluated in the present study, the heterogeneity observed in the occurrence of electrolyte disturbances may be partially related to interindividual differences in exposure to calcineurin inhibitors. Recent evidence has demonstrated that genetic variants, particularly the CYP3A5*3 polymorphism, significantly influence tacrolimus pharmacokinetics in hematopoietic cell transplantation recipients. *CYP3A5* encodes an enzyme involved in tacrolimus metabolism, and the CYP3A5*3 variant is associated with reduced or absent enzyme expression, resulting in decreased drug metabolism and increased systemic exposure to tacrolimus (Da Costa-Junior et al., 2025). Considering that calcineurin inhibitor-induced nephrotoxicity and tubular dysfunction are important mechanisms underlying the development of electrolyte abnormalities, it is plausible that pharmacogenetic factors may also contribute to individual susceptibility to these complications. Future studies incorporating pharmacogenetic markers may help identify patients at increased risk of developing immunosuppression-related electrolyte disturbances and support more individualized therapeutic strategies.

The findings of the present study have important clinical implications for the management of patients undergoing allogeneic HCT. Rather than representing isolated laboratory abnormalities, electrolyte disturbances should be recognized as dynamic and interrelated complications that may reflect the combined effects of calcineurin inhibitor therapy, kidney dysfunction, conditioning-related toxicity, and transplant-associated complications. The high prevalence of electrolyte disturbances observed during the early post-transplant period, together with their independent associations with acute kidney injury and with other electrolyte abnormalities, reinforces the need for systematic laboratory surveillance and early recognition of these alterations (Sureda et al., 2024). Current recommendations for the care of HCT recipients advocate close monitoring of serum electrolytes and renal function during the post-transplant period, particularly in patients receiving calcineurin inhibitors, to allow timely intervention and prevent potentially severe cardiovascular, neurological, and metabolic complications. In addition, recent cardio-oncology recommendations emphasize that prompt identification and correction of electrolyte abnormalities are essential strategies to reduce the risk of cardiac arrhythmias in HCT recipients (Gent et al., 2024; Kawtharany et al., 2026). Therefore, our findings provide additional evidence supporting a proactive approach to electrolyte monitoring and management during the first weeks after allogeneic HCT, which may contribute to improved supportive care and more individualized patient management.

Despite the relevance of the findings, some limitations should be acknowledged. First, the occurrence of arrhythmias associated with electrolyte disturbances could not be accurately assessed due to the lack of standardized documentation in the medical records. Second, potential changes in transplantation protocols over the study period, including conditioning regimens, graft-versus-host disease prophylaxis, and supportive care strategies, were not considered and may have influenced the occurrence of electrolyte disturbances and acute kidney injury.

Another limitation of this study is the incomplete availability of data on concomitant medications that may influence electrolyte homeostasis. Although the associations between cyclophosphamide and hyponatremia, as well as amphotericin B and hypokalemia, were evaluated, neither showed a significant association in the univariate analyses and were therefore not included in the multivariable models, according to our predefined statistical approach. However, information on other medications known to affect electrolyte balance, such as diuretics and proton pump inhibitors, was not systematically available because of the retrospective design of the study. Consequently, the potential influence of these agents as residual confounders cannot be excluded. Furthermore, relevant biochemical parameters that could have contributed to a more comprehensive understanding of the mechanisms underlying electrolyte disturbances, including parathyroid hormone, vitamin D, phosphate, and urinary electrolyte excretion, were not consistently available. Future prospective studies incorporating these variables may help further elucidate the complex pathophysiology of electrolyte disorders following allogeneic HCT.

In conclusion, this study demonstrate that electrolyte disturbances in patients undergoing HCT are frequent, multifactorial, and clinically relevant, arising from the combined effects of calcineurin inhibitors and transplant-related complications. The associations among dysnatremias and potassium and magnesium abnormalities, together with their cardiovascular and metabolic consequences, support an integrated pathophysiological interpretation of these disturbances rather than considering them in isolation. Although pharmacogenetic factors were not evaluated, emerging evidence suggests that genetic variability affecting calcineurin inhibitor exposure may contribute to interindividual susceptibility to electrolyte disturbances. Overall, these findings emphasize the importance of rigorous electrolyte monitoring and early intervention to improve therapeutic safety and clinical outcomes in patients undergoing HCT.

## Data Availability

The raw data supporting the conclusions of this article will be made available by the authors, without undue reservation.
